# Soft Skills in the Training of Future Dentists: Perceptions and Perspectives of Final-Year Students

**DOI:** 10.3390/dj14090552

**Published:** 2026-09-02

**Authors:** Cristina Gena Dascalu, Magda-Ecaterina Antohe, Diana Tatarciuc, Roxana Ionela Vasluianu, Cristina Iordache, Anca Vitalariu, Odette Elena Luca, Georgeta Liliana Foia

**Affiliations:** 1 Department of Medical Informatics and Biostatistics, Faculty of Medicine, “Grigore T. Popa” University of Medicine and Pharmacy, 16 Universității Street, 700115 Iasi, Romania; cristina.dascalu@umfiasi.ro; 2 Department of Implantology, Removable Dentures, Technology, Faculty of Dental Medicine, “Grigore T. Popa” University of Medicine and Pharmacy, 16 Universității Street, 700115 Iasi, Romania; roxana.vasluianu@umfiasi.ro (R.I.V.); marina.iordache@umfiasi.ro (C.I.); anca.vitalariu@umfiasi.ro (A.V.); elena-odette.luca@umfiasi.ro (O.E.L.); 3 Department of Medical Semiology, Faculty of Medicine, “Grigore T. Popa” University of Medicine and Pharmacy, 16 Universității Street, 700115 Iasi, Romania; diana.tatarciuc@umfiasi.ro; 4 Department of Dento-Alveolar and Maxillo-Facial Surgery, “Grigore T. Popa” University of Medicine and Pharmacy, 16 Universității Street, 700115 Iasi, Romania; georgeta.foia@umfiasi.ro

**Keywords:** soft skills, dentistry, dental students, empathy, professional motivation, cluster analysis, dental education, career choice, curriculum development

## Abstract

The practice of contemporary dentistry requires not only technical skills but also the development of soft skills, such as communication, empathy, and ethical and professional values. Although the importance of these skills is increasingly recognized, the perceptions of dental students in Romania regarding their relevance to their future professional practice remain insufficiently explored. **Objectives**: The study aimed to assess the perceptions of final-year students at the Faculty of Dentistry in Iași regarding the importance of soft skills, in relation to their demographic characteristics, professional motivations, and preferences regarding their future practice settings, as well as to identify distinct profiles through cluster analysis. **Material and Methods**: The study included 205 students in their fourth through sixth years at the Faculty of Dentistry in Iași, who anonymously completed a 26-item questionnaire rated on a Likert scale from 1 to 5. The statistical analysis included the chi-square test, Ward’s method, and k-means clustering. **Results**: Ethical and professional values, artistic skills, and empathy were most frequently rated as “very important.” Female students assigned significantly higher scores than male students to empathy, ethical and professional values, personal characteristics, and communication (*p* < 0.05–0.001). Professional independence, financial stability, and professional prestige were the main motivations for career choice. Cluster analysis identified four distinct profiles, with significant differences based on gender, professional motivations, and preferences regarding future practice. **Conclusions**: Students place a high value on soft skills, with differences based on gender and professional profile. The results support the integration of these skills into university education, to complement specialized clinical training.

## 1. Introduction

The practice of modern dentistry goes beyond the strictly technical aspects of dental care, incorporating effective communication, empathy, and ethical responsibility as fundamental pillars of professional conduct. Beyond the rigor of clinical skills, the quality of care is also defined by the dentist’s ability to build a therapeutic relationship based on respect, trust, and an understanding of each patient’s unique circumstances [1,2,3]. The set of these cross-cutting skills, known in the literature as “soft skills” (non-technical competencies), includes, among others, communication and interpersonal skills, ethical and professional values, personality traits, managerial ability, and empathy [4,5]. Although the role of these competencies in ensuring the quality of dental care is increasingly recognized internationally, research in the field highlights an uneven focus on the various dimensions of non-technical competencies. Scientific interest has focused primarily on intrapersonal skills, while ethical and professional values and competencies related to the organization and coordination of professional activity have been investigated to a considerably lesser extent, both among students and experienced dentists [2].

The focus on developing non-technical competencies within dental education emerged at the European level as early as 2009, when it was explicitly stated that the training of future dentists should include, alongside clinical competencies, the development of social, interpersonal, and communication skills. Subsequently, this perspective has gained increasing relevance within relevant higher education institutions in North America [6,7,8]. Although the importance of non-technical competencies in the training of future dentists is increasingly recognized, their inclusion and integration into university curricula vary considerably among educational institutions, and their assessment remains challenging due to the predominantly subjective nature of the available assessment tools and the absence of standardized curricular frameworks [9,10,11]. Empathy occupies a special place among non-technical competencies; it is defined as the ability to perceive and understand a patient’s feelings, situation, and problems without becoming emotionally entangled with them. Studies conducted using validated instruments, such as the Jefferson Empathy Scale, consistently show statistically significant differences between genders, with women scoring higher than men among both medical and dental students [12,13,14]. This phenomenon was also confirmed among Thai dental students, where the reported level of empathy varied significantly by gender and year of study [15]. Professional empathy contributes to increased patient satisfaction, reduced anxiety and discomfort experienced in the dental office, and, consequently, improved treatment compliance [16,17], which is why assessing and cultivating it throughout the years of study is a relevant educational objective.

The choice to pursue a career as a dentist is, in turn, a process driven by multiple factors, which vary from country to country depending on the socioeconomic and cultural context. Studies conducted in Ireland and Saudi Arabia converge in highlighting professional independence, financial stability, flexible working hours, and the opportunity to help people as the main motivations for choosing this career [18,19,20]. A recent study conducted among Irish dental students identified altruism and favorable working conditions as the main motivational factors, alongside previous positive experiences related to the dental profession, while also highlighting the influence that nationality, admission pathway, and year of study have on these motivations [4]. Understanding these factors is relevant not only from an educational perspective but also for anticipating future preferences regarding the professional practice environment.

Although soft skills are receiving increasing attention in the relevant international literature, how dental students in Romania perceive their relevance to future professional practice remains insufficiently explored. This study aims to analyze how students in their final years at the Faculty of Dentistry within the “Grigore T. Popa” University of Medicine and Pharmacy in Iași perceive the importance of various categories of soft skills—cognitive, communication and interpersonal, artistic, ethical-professional, personal, and managerial, as well as empathy—for their future practice as dentists, in relation to their demographic characteristics, the motivations behind their choice of this career, and their preferences regarding their future practice setting. A secondary objective of the research was to identify, through multivariate cluster analysis, distinct student typologies based on their profile regarding the value they place on these competencies—information that can help inform the development of differentiated educational strategies within the dental curriculum.

## 2. Materials and Methods

Participants/Data collection: Data collection took place between February and April 2025, among students enrolled in the 4th, 5th, and 6th years of study at the Faculty of Dental Medicine, “Grigore T. Popa” University of Medicine and Pharmacy from Iași, Romania. The eligible population comprised 362 students: 72 in the 4th year (distributed across 3 groups of 24 students each), 150 in the 5th year, and 140 in the 6th year. Of these, 205 students completed the questionnaire, corresponding to a response rate of 56.6% (205/362). The only exclusion criterion applied was incomplete questionnaire submission: 20 students started the questionnaire but did not finish it, so these responses were tagged as invalid and excluded from the analyzed sample. The other remaining 137 eligible students did not access the questionnaire and were therefore identified as non-respondents.

Students were asked to anonymously fill out a 26-item questionnaire in which they expressed their level of agreement with different statements regarding the use of soft skills in the dental office, during the interaction with the patient; the agreement level was expressed using a Likert scale from 1 to 5, where 1 corresponded to total disagreement and 5 to total agreement. The students were also asked to indicate the motivations that led them to become stomatologists, their preferences regarding the future practice, and their opinions about supplementary knowledge they need in order to become good practitioners. The questionnaire was presented and explained separately to each subject, along with the research goals.

Questionnaire: The questionnaire was adapted from a questionnaire developed within the framework of the Erasmus+ project “Exploring, Assessing and Applying SOFT skills in DENTAL Medicine University students” (DENTA_SOFT), no. 2022-1-BG01-KA220-HED-000085701 [21]. Based on models already existing in the scientific literature, the questionnaire was the outcome of a collaborative development process among the partner institutions of the DENTA_SOFT consortium—our faculty included—and was finalized by the oral-health-education experts at the coordinating institution, the University of Bulgaria. Item proposals were contributed by each partner country’s team, drawing on national experience and perspectives on non-technical competencies in dental education. Rather than a single source version being translated uniformly, the adaptation to each national context—including the Romanian one—was carried out separately by the respective partner team, with particular attention to themes of relevance to contemporary professional practice, notably the interplay between digitalization and the role of soft skills in dentistry.

The questionnaire’s clarity and relevance were assessed by a team of international specialists by calculating the IRA (inter-rater agreement), ICV (item content validity), and SCV (scale content validity) indexes, and its internal consistency was assessed by calculating the Cronbach’s alpha index (0.724); on these bases, the questionnaire was considered suitable for being applied in our study. Prior to the wide-scale administration, the questionnaire was first administered to 25 students as a pilot phase. Among these students, 14 obtained low or very low scores regarding their levels of soft skills; these students were subsequently invited to attend dedicated soft skills training sessions organized as part of the project, as a form of educational follow-up. The students who participated in the pilot phase were also included in the final sample of 205 respondents.

Variables: The questionnaire was made up of 20 items aiming to evaluate 6 different types of soft skills: cognitive skills (3 items), communication and interpersonal skills (7 items), artistic skills (1 item), ethical and professional skills (5 items), personal features (2 items), and management skills (2 items). Based on these items, 6 different scores were calculated, each one corresponding to the importance assigned by the students to a specific category of soft skills. One of the questionnaire’s items made direct reference to the stomatologist’s level of empathy in his relation with patients, as a 7-th soft skill mandatory for good practice. Other variables included in the study were the students’ motivations to become stomatologists, their preferences for practice and their opinions about supplementary, but necessary knowledge, as well as their basic demographic features (gender and age).

Statistical analysis: The data from the questionnaire were recorded in a data file in SPSS 31.0 (SPSS Inc., Chicago, IL, USA) for Windows. The answers to each item were characterized through frequency distributions and contingency tables. The numerical variables were characterized through descriptive statistics (mean, standard deviation, range, median, and IQR). The comparisons between samples were performed using the Chi-squared test (with Fisher correction when necessary). We considered the *p* < 0.05 value as statistically significant (*) and the *p* < 0.01 value as statistically highly significant (**); the *p*-values between 0.05 and 0.1 were also highlighted as borderline statistically significant. In order to investigate the students’ typologies (if any) based on these categories of soft skills importance scores, we first performed a hierarchical cluster analysis using Ward’s method and squared Euclidean distance (based on the corresponding Z-scores). The optimal number of clusters was determined using the dendrogram and the agglomeration coefficients, after which k-means clustering was applied in order to refine the cluster structure. The Z-scores were interpreted according to the following criteria: values > +0.5 = high importance (above average); values between −0.5–0.5 = average importance; values < −0.5 = low importance (below average).

Ethical statement: Participation in our study was voluntary. The subjects were informed about the study and the content of the questionnaire, and they agreed to participate by providing their informed consent. The questionnaires were completed anonymously in order to protect the subjects’ privacy and obtain as many objective answers as possible. The study was approved by the Ethical Committee of “Grigore T. Popa” University of Medicine and Pharmacy from Iași, Romania (decision no. 427/7 April 2024).

## 3. Results

### 3.1. General Overview of the Group of Students

Two-thirds of the students (66.3%) are Romanian, and approximately two-thirds (63.4%) are female; half of the students (54.6%) are aged between 23 and 24 years; nearly one-third (29.8%) are under 22 years old, the remainder being over 25 years old.

First, the students were asked about the motivations that led them to choose the profession of dentist (Table A1). The vast majority of students (82.4%) stated that what primarily motivated them to choose this profession was professional independence and flexible working hours; the next argument, cited by 70.7% of students, was financial stability, followed by professional prestige (67.8% of students). While 58.0% of students think that dentistry is a caring and noble profession, 27.8% chose this career because it offers many social opportunities, and 23.9% of students like to work as part of a team. With regard to students’ opinions on additional competencies they would need beyond their core specialty subjects (Table A1), the vast majority (83.9%) indicated medical ethics; 76.1% of students considered knowledge in healthcare management and marketing necessary; other competencies highlighted by the students were legislation (73.7%), dental ergonomics (71.7%) and behavioral sciences (68.3%). In terms of students’ preferences for where to practice their profession after graduation (Table A1), three-quarters (75.1%) stated that they wish to work in a large dental clinic, and half (52.2%) stated that they wish to work in a single-dentist practice; 31.7% of students also mentioned a career in university teaching or research as a possible option.

Regarding students’ opinions on the importance of various soft skills for the practice of dentistry, the following was observed across the entire study sample: 21.0% of students considered cognitive skills to be very important for the practice of dentistry, while 22.4% of students evaluated communication and interpersonal skills in the same way; in contrast, more than half (56.1%) consider artistic skills to be very important, and 61.0% consider ethical and professional values to be equally important. Personal characteristics are considered to be very important by 37.6% of the students, and management skills by 27.3%. On the other hand, 59.0% of students believe that empathy is of very great importance in the practice of dentistry, and another 18.5% consider this trait to be of great importance (Table 1). A quantitative breakdown of the calculated scores is presented in Table 2.

### 3.2. Univariate Analysis

In the first stage, we conducted a comparative study of the degree of importance students attach to soft skills based on their demographic characteristics.

The comparative study by gender highlights statistically significant differences in the case of empathy, which is considered very important by 67.7% of girls compared to only 44.0% of boys; ethical and professional values were considered very important by 70.8% of girls compared to 44.0% of boys; personal characteristics were considered very important by 44.6% of girls compared to only 25.3% of boys; and communication and interpersonal skills were considered very important by 29.2% of girls compared to only 10.7% of boys. Other types of skills were also considered very important by more of the girls surveyed than by the boys, but the differences were not large enough to reach the threshold for statistical significance (Table A2).

The comparative study between Romanian and international students reveals statistically significant differences only in the case of ethical and professional values, which are considered very important by 68.4% of Romanian students compared to 46.4% of international students, and in management skills, which are considered very important by 49.3% of Romanian students compared to 17.4% of foreign students. Foreign students place a very high importance on cognitive skills in 27.5% of cases, compared to only 17.6% of Romanians, while Romanian students more frequently than foreign students place very high importance on communication and interpersonal skills (in 24.3% of cases compared to 18.8%) and personal characteristics (in 41.2% of cases compared to 30.4% of foreign students). Furthermore, empathy is considered very important by 64.0% of Romanian students compared to 49.3% of foreign students, a difference that is at the threshold of statistical significance (Table A3).

No statistically significant differences were found among the three age groups identified with regard to the importance of soft skills. It must be noted, however, that students over 25 years of age more frequently than others attach great importance to cognitive skills (in 28.1% of cases), communication and interpersonal skills (in 31.3% of cases), as well as artistic skills (in 68.8% of cases) and empathy (in 62.5% of cases). Empathy is, however, also considered very important among the other age groups, by 57.4% of students aged 22 and under and 58.9% of those aged 22 to 23. Ethical and professional values are considered very important by 64.3% of students aged 23 to 24, while personal characteristics and management skills are most frequently rated as very important in the age group up to 22 years old: 41.0% of students in this category consider personal characteristics to be very important, and 36.1% rate management skills similarly (Table A4).

We also conducted a comparative assessment of the importance of soft skills in relation to the reasons that led students to choose a career in dentistry (Table A5), the additional skills they consider necessary for the most comprehensive professional training (Table A6), and their preferences regarding where to practice in the future (Table A7).

We found no statistically significant differences between students who chose the dental profession because they consider it prestigious and respectable (M1) and the others in terms of the importance they attach to the cross-cutting competencies investigated. However, students in the first category more frequently than others attach very high importance to cognitive competencies (in 23.7% of cases compared to 15.2%), while students who did not were guided by the criteria of prestige and respectability in choosing their profession more frequently than others attach great importance to communication and interpersonal skills (in 27.3% of cases versus 20.1%) and personal characteristics (in 40.9% of cases versus 36.0%). The other types of transferable skills are rated almost similarly, including empathy, which is considered very important by 59.7% of students motivated by professional prestige, compared to 57.6% of the others.

Students who chose the dental profession for the opportunity to work as part of a team (M2) are more likely than others to place a very high value on cognitive skills (in 30.6% of cases compared to 17.9%), as well as on ethical and professional values (in 67.3% of cases compared to 59.0%) and personal characteristics (in 46.9% of cases compared to 34.6%). Empathy is considered very important by these students in 61.2% of cases, a percentage close to that reported by the others (58.3%). None of these discrepancies are statistically significant.

No statistically significant differences were found regarding the opinions of students who chose to become dentists because of the opportunity to be professionally independent and have flexible working hours (M3) compared to the others. Students in this category are less likely than others to place a very high importance on soft skills—a fact that is particularly noticeable in the case of cognitive skills (rated as very important by only 20.1% of students in this category compared to 25.0% of the others), ethical and professional values (rated as very important by 58.6% of students in this category compared to 72.2% of the others), personal characteristics (rated as very important by 35.5% of students in this category compared to 47.2% of the others), and management skills (rated as very important by 26.6% of students in this category compared to 30.6% of the others). Empathy, on the other hand, is rated almost similarly across the two groups of students, being considered very important by 58.6% of students in the first category and by 61.1% of the others.

However, several statistically significant differences were found between students who chose the dental profession for financial stability (M4) and the others. It is interesting to note that these students attach great importance to cognitive skills in only 15.9% of cases, a percentage that doubles to 33.3% among students whose career choice is not motivated by financial stability; a similar situation is observed for management skills, which are also rated as very important in 24.1% of cases, compared to 35.0%; in both cases, the differences are statistically significant. The other skills in the soft skills category are also considered less important by students in the first category compared to the others, although the discrepancies are smaller and do not reach the threshold for statistical significance—this is the case for artistic skills (considered very important in 53.1% of cases compared to 63.3%), ethical and professional values (considered very important in 56.6% of cases compared to 71.7%), and personal characteristics (considered very important in 33.8% of cases compared to 46.7%). Empathy is considered very important in similar percentages across the two categories of students.

In general, there are no statistically significant differences between the opinions of students who chose the dental profession because they consider it a humane and noble one (M5) and those of the others, with one exception: personal characteristics, which are considered very important by 42.0% of students in this category compared to 31.4% of the others. In fact, the other soft skills are also considered very important more frequently among students in this category compared to the others, even though the differences do not reach the threshold for statistical significance. This is the case for cognitive skills—considered very important by 22.7% of students motivated by the nobility and humanity of the dental profession compared to 18.6% of the others—as well as for artistic skills (58.8% versus 52.3%), for ethical and professional values (63.9% versus 57.0%), and even for management skills (29.4% versus 24.4%). Empathy is considered very important by 58.0% of students in this category, compared to 60.5% of the others.

A final group of students chose to become dentists because they believe the profession offers them numerous social interactions (M6). But also in this case, no statistically significant differences were found compared to the others regarding their views on the importance of soft skills. Students motivated by social interactions are more likely than others to place a very high importance on the following categories of transferable skills: communication and interpersonal skills (in 31.6% of cases compared to 18.9% among the others—a difference close to the threshold of statistical significance), artistic skills (in 66. 7% of cases compared to 52.0%), ethical and professional values (in 68.4% of cases compared to 58.1%), personal characteristics (in 42.1% of cases compared to 35.8%), and, in addition, empathy (in 63.2% of cases compared to 57.4%). In contrast, these students place less importance on cognitive skills, which are rated as very important by only 15.8% of them, compared to 23.0% of the others.

Students who felt they needed additional knowledge of medical ethics (C1) were also more likely than others to consider the following categories of soft skills to be very important: cognitive skills (in 21.5% of cases compared to 18.2%), artistic skills (in 57.0% of cases compared to 51.5% among the others), ethical and professional values (in 62.2% of cases compared to 54.5% among the others), and personal characteristics (in 40.1% of cases compared to 24.2% among the others). Students in this category, however, place less importance than others on communication and interpersonal skills, which are considered very important in 20.9% of cases compared to 30.3% among the others. Empathy is considered very important in 59.3% of cases, a percentage very close to that recorded in the other category (57.6%). None of these differences, however, reach the threshold for statistical significance.

Students who felt they needed additional knowledge of health legislation (C2) were also more likely than others to consider the following types of soft skills to be very important: communication and interpersonal skills (in 23.8% of cases compared to 18.5% among the others), as well as artistic skills (in 58.3% of cases compared to 50.0%), ethical and professional values (in 64.2% of cases compared to 51.9%), personal characteristics (in 39.7% of cases compared to 31.5%), and last but not least, empathy, in 61.6% of cases compared to 51.9%. Cognitive skills are considered very important by only 18.5% of these students, compared to 27.8% of the others, and management skills are rated similarly by students in this category compared to the others.

Students who believe they need knowledge in the field of behavioral sciences (C3) place a very high importance on ethical and professional values in 66.4% of cases, compared to 49.2% of the others, and on personal characteristics in 42.9% of cases, compared to 26.2% of the others—both differences being statistically significant. Likewise, these students place a very high importance on communication and interpersonal skills more frequently than others (in 26.4% of cases compared to 13.8% of others), artistic skills (in 58.6% of cases compared to 50.8%), and empathy (in 62.9% of cases compared to 50.8%). In contrast, they place less importance on cognitive skills (considered very important in only 19.3% of cases compared to 24.6% among other students) and management skills (considered very important in only 25.0% of cases compared to 32.3% among other students).

Students who believed that knowledge of health care management and marketing (C4) was necessary for them do not differ statistically significantly from the others in terms of the importance they attach to soft skills. However, they are more likely than others to place a very high importance on ethical and professional values (in 62.2% of cases versus 57.1%) and, naturally, on management skills (in 28.2% of cases versus 24.5%). These same students, on the other hand, place less importance on cognitive, communication, and interpersonal skills, as well as artistic skills and even empathy, which is considered very important by 58.3% of these students compared to 61.2% of the others.

Several statistically significant differences were found regarding the importance placed on soft skills among the group of students who felt they needed additional knowledge of dental ergonomics (C5) compared to the others. Students in this category place a very high importance on personal characteristics significantly more often than the others (in 42.2% of cases compared to 25.9% among the others) and place a very high importance on management skills significantly less often than the others (in 25.9% of cases compared to 31.0% among the others). Students in this category also place a very high importance on artistic skills more frequently than others (in 58.5% of cases compared to 50.0%) and on ethical and professional values (in 63.9% of cases compared to 53.4% among the others). Empathy is considered very important by 58.5% of students in this category, compared to 60.3% of the others—thus, similar percentages.

Students who wish to work in a single-dentist practice (P1) generally place less importance on soft skills compared to those who did not express this preference; in some cases, the differences are even statistically significant—this is the case for cognitive skills, which are considered very important by 13.1% of these students compared to 29.6% of the others, and for ethical and professional values, considered very important by 54.2% of these students compared to 68.4% of the others. Only management skills are considered very important by nearly equal percentages of students in this category (28.0%) compared to the others (26.5%). Empathy is considered very important by 55.1% of students in this category compared to 63.3% of the others.

Students who expressed a preference for working in a large dental clinic (P2) place a very high value on artistic skills in 61.0% of cases, compared to 41.2% of students in the other category—a statistically significant difference. They also more frequently place very high importance on ethical and professional values (in 64.3% of cases compared to 51.0% of the others) and personal characteristics (in 40.3% of cases compared to 29.4% of the others). In contrast, these same students place less importance than the others on management skills and even empathy, which is considered very important by 57.8% of them compared to 62.7% of the others.

Students who expressed a preference for pursuing an academic career (P3) are statistically significantly more likely than others to place a very high importance on cognitive skills (in 36.7% of cases compared to 16.0% of the others); they also more frequently attach great importance to artistic skills (in 69.4% of cases compared to 51.9% of the others), ethical and professional values (in 71.4% of cases compared to 57.7% among the others), and empathy (in 65.3% of cases compared to 57.1% among the others)—however, the differences in these cases do not reach the threshold for statistical significance. Communication and interpersonal skills, personal characteristics, and management skills are rated almost similarly by these students compared to those who do not wish to pursue a career in academia.

A small number of students also expressed an interest in a career in research. They do not differ significantly from the others in terms of the importance they place on soft skills; however, it is worth noting that a higher percentage of them place a very high importance on cognitive skills (37.5% compared to 19.6% of the others), ethical and professional values (68.8% compared to 60.3% of the others), and personal characteristics (43.8% compared to 37.0% of the others). Communication and interpersonal skills are considered less important by students in this category than by the others, as are management skills and even empathy, which was considered very important by 56.3% of them compared to 59.3% of the others.

### 3.3. Multivariate Analysis

First, we performed the hierarchical clustering of the corresponding Z-scores for the six investigated categories of soft skills; we inspected the Agglomeration Schedule in order to identify the significant changes; a substantial increase in the fusion coefficients was detected at the final stages of the clustering process (stage 203–204), suggesting a four-cluster solution as the most appropriate representation of the data structure. We then ran the k-means cluster analysis (for four clusters) in order to stabilize the solution and characterize it more accurately.

**Figure 1:**
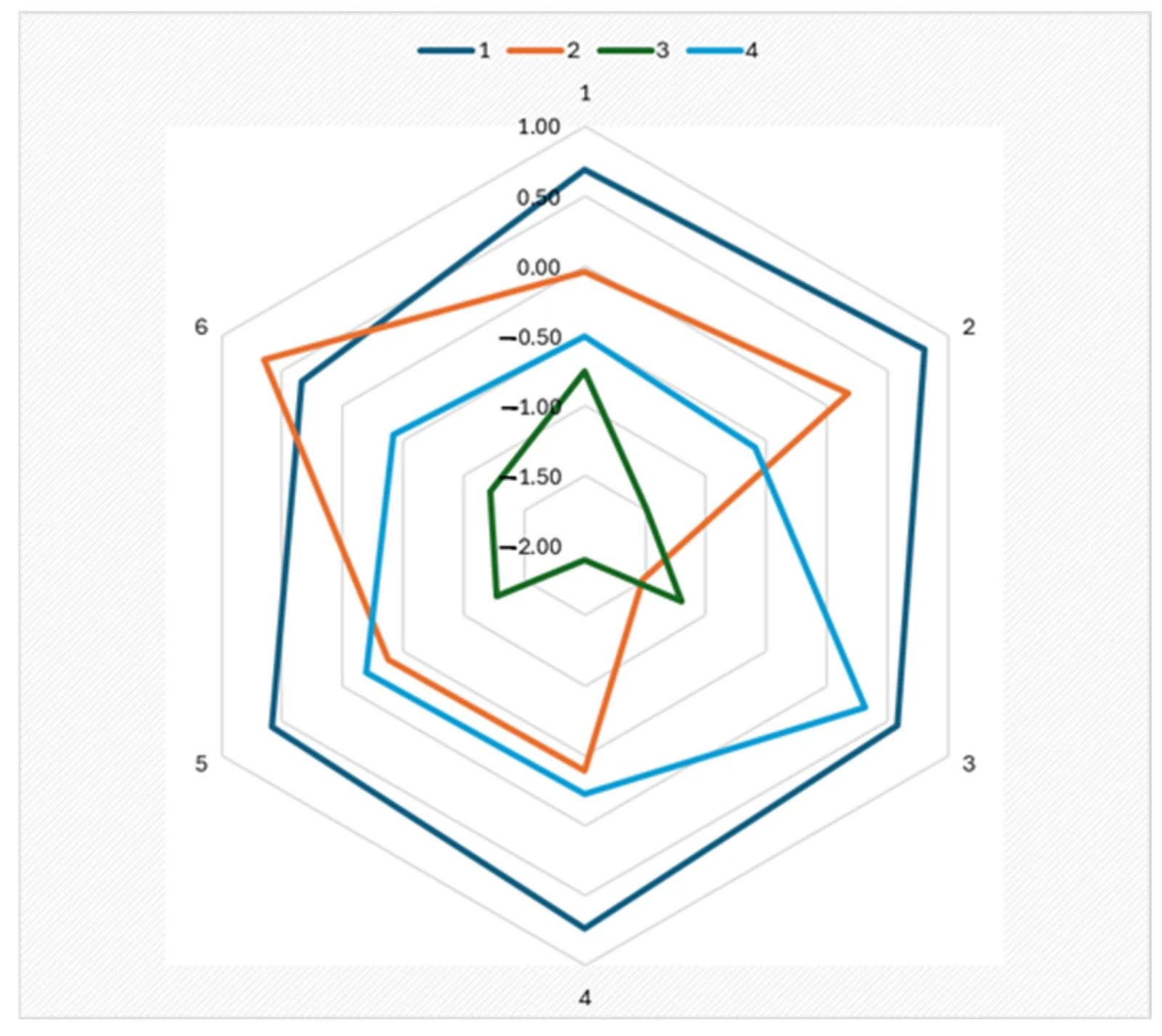
The visual characterization of the identified clusters (1–4) according to the Z-scores of the 6 soft skills categories (1—cognitive skills; 2—communication skills; 3—artistic skills; 4—ethical-professional values; 5—personal features; 6—management skills).

The first cluster comprises 75 students; they have a high overall profile, placing a high value on nearly all types of competencies examined. The competencies considered most important are communication and interpersonal skills (0.802), followed by ethical and professional values (0.737) and cognitive skills (0.690). The only category of competencies with an average importance score is management (0.342). The second cluster comprises 36 students, who have a pragmatic-managerial profile. They consider management competencies to be the most important (0.653), which indicates a strong practical orientation; cognitive and communication competencies, ethical and professional values, and personal characteristics are considered of moderate importance, while artistic competencies are considered the least important (−1.526). The third identified cluster comprises only 12 students, who consider all six categories of competencies investigated to be of low importance (below average). These opinions may indicate significant difficulties for the students in question, which may require specific educational interventions. The fourth cluster identified also includes a large group of 82 students with a moderately creative profile—they place the greatest importance on artistic skills (which, however, fall within the average range, 0.319). A moderate, although lower, degree of importance is also assigned to personal characteristics, ethical and professional values, and management skills, while communication and cognitive skills are rated below average—these opinions indicate a relatively balanced personal profile, even though students in this group do not seem to place a high value on academic performance (Table 3, Figure 1).

Statistically significant differences were found among the four identified clusters in terms of gender composition. Thus, in the first two clusters—the one with a high global profile and the one with a pragmatic-managerial profile—females predominate (76.0% in the first cluster and 69.4% in the second, respectively), while in the third cluster, with a low global profile, boys predominate (75.0%), and the fourth cluster, with a moderate-creative profile, has a relatively balanced gender distribution (45.1% male and 54.9% female). However, no statistically significant differences were found in terms of distribution by age group. The largest proportion of students over 25 years of age (18.7%) was identified in the cluster with a high global profile, which, however, also contains the largest proportion of students under 22 years of age (32.0%); the cluster with a pragmatic-managerial profile and the one with a low global profile have the highest proportions of students aged 23 to 24 (over 60%). Furthermore, no statistically significant differences were found regarding the students’ nationality. It is evident that Romanian students are in the majority, but the highest proportion of international students—50.0%—was observed in the cluster with a low global profile, followed by the cluster with a pragmatic-managerial profile, which includes 41.7% international students (Table 4).

A comparative study of the motivations for choosing a career in dentistry expressed by students in the four clusters reveals the following: most students who chose dentistry because it is a prestigious and respectable profession belong to the cluster with a pragmatic-managerial profile (72.2%), while the fewest belong to the cluster with a low global profile (50.0%); Similarly, most students whose primary motivation was the desire to work as part of a team belong to the cluster with a high global profile (32.0%), while only 8.3% of those in the cluster with a low global profile expressed the same preference. In contrast, most students whose primary motivation was the desire to be self-employed and have flexible working hours belong to the low-global-profile cluster (91.7% of them), and the fewest (77.3%) belong to the high-global-profile cluster. Most students who chose this profession for financial stability belong to the moderate-creative-profile cluster (80.5%), while the fewest belong to the high-global-profile cluster (58.7%)—this is, in fact, the only statistically significant difference identified among the four clusters. Most students who chose dentistry because it is a humane and noble profession belong to the cluster with a high global profile (64.0%), followed by the cluster with a moderate-creative profile (59.8%), while the fewest students with this motivation were found in the cluster with a low global profile (33.3%). Similarly, most students who chose dentistry because it offers numerous social contacts also belong to the cluster with a high global profile (34.7%), while the fewest (8.3%) belong to the cluster with a low global profile (Table 5).

58.3% of students in the low-global-profile cluster identified medical ethics as an additional subject they need for their dental practice, in contrast to over 80% of students in the other three clusters—a discrepancy that is quite pronounced, falling just below the threshold for statistical significance. Similarly, only 33.3% of students in the low-global-profile cluster indicated that health legislation is necessary for their future practice, compared to 75–80% of students in the other clusters; 33.3% of students in this cluster also indicated behavioral sciences as an additional subject of study necessary for their future practice, a percentage that rises to 60–70% among students in the other clusters—the two differences thus recorded between this cluster and the others are statistically significant. On the other hand, nearly all students (91.7%) in the cluster with a low global profile indicate health management and marketing as a needed additional field of study, compared to less than 80% of students in the other clusters. Finally, most students who consider dental ergonomics to be necessary belong to the high-overall-profile cluster (78.7%), while the fewest again belong to the low-overall-profile cluster (50.0%)—however, the differences observed in these cases do not reach the threshold for statistical significance (Table 5).

Approximately 40% of students in the cluster with a high global orientation, as well as those in the cluster with a low global orientation, wish to practice their profession after graduation in a single dental practice, compared to 80.6% of those in the cluster with a pragmatic-managerial orientation, which thus differs statistically significantly from the others. Similarly, 80% of students in the high-global-profile cluster or the moderate-creative-profile cluster wish to practice their profession in a large dental clinic, compared to only 52.8% of students with a pragmatic-managerial profile—who thus opt for an independent career—and 66.7% of students with a low-global-profile cluster —again, a highly statistically significant difference. An academic career is preferred most frequently by students in the high-global-profile cluster (32.0% of them) and least frequently by students with a pragmatic-managerial profile (13.9% of them) or a low-global-profile cluster (16.7%). A career in research is generally unpopular; however, it was indicated by 10.7% of students in the high-global-profile cluster, compared to about 5% of those in the pragmatic-managerial or moderate-creative-profile clusters (Table 5).

There are also highly statistically significant differences among the four clusters regarding the importance students place on empathy in dental practice. Most students who consider empathy to be very important belong to the cluster with a high global profile (76.0%), followed by the cluster with a pragmatic-managerial profile (72.2%)—which differs fundamentally from those in the cluster with a moderate-creative profile, of whom only 43.9% share this view, and especially from those in the low-global-profile cluster, where the corresponding percentage is only 16.7%. Overall, 25.0% of the students in this cluster believe that empathy is of very little importance in dental practice, and half (50.0%) consider its importance to be moderate (Table 6).

## 4. Discussion

The results of this study, conducted on a sample of 205 students in their final years at the Faculty of Dentistry in Iași, are largely consistent with the trends highlighted in the international specialized literature regarding students’ perceptions of the relevance of soft skills to their future professional practice.

Regarding career choice motivations, professional independence and flexible work schedules, financial stability, and professional prestige ranked at the top of the list of reasons cited by the students surveyed. These results are consistent with those reported in other countries, where professional autonomy, job security, and favorable financial prospects are the main reasons underlying the choice of a career in dentistry [4,6]. Unlike studies in which altruism is highlighted as one of the main reasons for choosing a career [4], in our study, the desire to help others—expressed through the option “a humane and noble profession”—occupied a secondary position in the hierarchy of motivations. This difference could be associated with the particularities of the current cultural and socioeconomic context and may also reflect trends observed in other international contexts, where economic considerations are playing an increasingly important role in professional career choices [4]. The findings regarding gender differences are also consistent with the literature: female students participating in the study placed significantly greater importance than male students on empathy, ethical and professional values, personal characteristics, and communication and interpersonal skills. This pattern mirrors findings from international studies that use validated empathy assessment tools, such as the Jefferson Scale, and that consistently report higher scores among female students in both medical and dental schools [5,8]. Explanations put forward in the literature for these differences include both the influence of socialization and learning processes and the possible contribution of biological factors, although their relative importance has not been fully elucidated [5]. From an educational perspective, the lower importance attributed to empathy and ethical-professional values by male students in the study sample highlights the need to strengthen these dimensions within the professional training process, through educational strategies tailored to the needs and characteristics identified among different student groups. A notable aspect of the results is the high importance students place on artistic skills and ethical and professional values, both of which were rated as “very important” by more than half of the respondents—a proportion higher than that recorded for cognitive or communication skills. This result can be linked to the nature of contemporary dental practice, in which the aesthetic component of treatments is becoming increasingly important, alongside the ever-stricter ethical requirements imposed by patients’ growing level of awareness. At the same time, the overwhelming preference among students for additional knowledge of medical ethics (83.9%) confirms the concern—also highlighted in recent literature—regarding the importance of strengthening the ethical and legal components of the dental curriculum [2].

Multivariate cluster analysis enabled the identification of four distinct student typologies, which differ significantly both in terms of their profile regarding the valuation of non-technical competencies and in terms of demographic structure, professional motivations, and preferences for future practice. The combined use of Ward’s hierarchical analysis, followed by refinement using the k-means method, is a commonly used methodological approach for identifying student typologies, as it allows both for the objective determination of the optimal number of clusters and for the stabilization of the final classification solution. It should be noted, however, that the validity of the results obtained using the k-means method depends on the fulfillment of certain assumptions regarding data distribution (relatively homogeneous, well-separated groups of comparable sizes), a factor that must be taken into account when interpreting and generalizing the identified typologies.

It is noteworthy that the third cluster identified—characterized by a low overall valuation of all categories of non-technical competencies—included a small but significant number of students (n = 12), predominantly male, who placed little importance on empathy, professional ethics, or healthcare legislation. Although this group is a minority, it raises a red flag from an educational perspective, as it may reflect either a genuine gap in these students’ training or an underestimation of the relevance of non-technical competencies for their future professional practice—both hypotheses that warrant targeted educational interventions [1,2].

In terms of preferences regarding future practice settings, it is noteworthy that students who are oriented toward an academic career placed significantly greater importance on cognitive skills—a finding consistent with the profile of this career orientation— while students who prefer to practice in a private practice generally placed less value on non-technical skills, including empathy. This latter observation warrants further investigation, as private practice typically involves direct and intensive contact with patients, a context in which empathy and communication skills play an essential role [2,7].

Beyond their descriptive value, the results have relevant implications for how future dentists will practice their profession. A first aspect concerns the professional training process: the declarative recognition of the importance of soft skills does not automatically imply their effective implementation in clinical practice. Data from the literature suggest that clinical experience, in the absence of a well-structured educational framework, does not in and of itself ensure the adequate development of soft skills. Developing these skills requires the deliberate integration of specific pedagogical strategies into the educational curriculum of future dentists, such as courses focused on the doctor–patient relationship, simulations, role-playing, and case-based learning. Such approaches can contribute to the gradual and sustainable strengthening of empathy and communication skills, facilitating their application in professional conduct and in the relationship with the patient [9,10]. In this regard, the results of the present study can serve as a starting point for developing dedicated modules integrated into the final years of study, when students are nearing their actual entry into practice and most acutely perceive the need for such competencies.

Another relevant aspect concerns students’ preferences regarding their future professional practice setting. A focus on clinical practice in a large dental clinic, organized around a multidisciplinary team, places particular emphasis on soft skills, especially interprofessional communication, collaboration, and conflict resolution. In such a professional context, the quality of dental care depends not only on individual clinical competence, but also on each professional’s ability to communicate effectively, coordinate their work with other team members, and contribute to building a professional environment based on cooperation and shared responsibility. Conversely, the generally lower importance attributed to empathy and communication skills by students who envision their future practice in a single-dentist practice has significant implications for professional practice. In such a setting, characterized by a higher degree of autonomy and more limited opportunities for collaboration and peer feedback, soft skills take on particular significance in building and maintaining a high-quality therapeutic relationship. Any shortcomings in the areas of empathy and communication can influence the patient’s experience, level of satisfaction, and the continuity of the relationship with the dentist—aspects of particular relevance in the context of single-dentist practice [2,7].

The motivations underlying career choices, in turn, have implications for future practice. Students who chose dentistry primarily for reasons of financial stability placed significantly less importance on both cognitive and managerial skills compared to their peers—a correlation that warrants monitoring over time, since motivation focused exclusively on financial aspects, without a parallel emphasis on organizational skills and the continuous updating of knowledge, is associated in the literature with an increased risk of burnout and lower long-term job satisfaction. Conversely, students motivated by the opportunity to work in a team or by the desire to help people demonstrated a more balanced appreciation of the range of non-technical skills—a profile that is expected to facilitate better adaptation to the interpersonal demands of daily practice.

The overwhelming demand expressed by students for additional knowledge in medical ethics, healthcare management and marketing, health legislation, and dental ergonomics effectively outlines a roadmap of continuing education needs for the years immediately following graduation. These results suggest the opportunity to introduce, as early as during their undergraduate studies, applied modules on dental practice management and professional ethics, which would prepare students for the administrative and ethical challenges of independent practice—from managing the contractual relationship with the patient and obtaining informed consent, to organizing workflow and adhering to ergonomic standards, which have a direct impact on the occupational health of future practitioners.

Last but not least, the identification of a minority group of students with a generally low valuation of non-technical skills, including empathy, has direct implications for patient safety and the quality of dental care: the absence of empathy and a solid ethical foundation is associated, in the clinical literature, with a higher risk of misunderstandings in the patient–physician relationship, lower treatment compliance, and consequently, a higher medico-legal risk [2]. For this group, some form of individualized mentoring or career counseling during their final years of study could be helpful, aimed at compensating, as much as possible, for this shortfall before they begin independent practice. Similarly, the consistent differences in favor of female students regarding empathy and ethical-professional values suggest that programs for developing non-technical skills could benefit from a gender-differentiated approach, with a special emphasis on strengthening these dimensions among male students, without, however, neglecting the specific developmental needs of any category of students.

The limitations of this study stem, first and foremost, from the cross-sectional nature of the research, which does not allow for causal conclusions, as well as from the fact that the sample consisted of students from a single educational institution, which limits the generalizability of the results to the entire population of dental students in Romania. At the same time, the self-reported nature of the responses constitutes an inherent limitation of this type of research, as the perceptions expressed by participants may be influenced by a tendency to provide answers that are considered socially acceptable, without always accurately reflecting the attitudes or behaviors exhibited in actual practice. The small size of certain subgroups analyzed—such as the cluster characterized by a low overall profile—also calls for caution in interpreting the identified differences. Future research, conducted in multicenter settings and based on a longitudinal design, could contribute to a better understanding of how perceptions of soft skills take shape and evolve throughout university education, as well as the role that targeted educational interventions can play in developing and strengthening these skills.

## 5. Conclusions

The present study highlights that the vast majority of students in their final years at the Faculty of Dental Medicine in Iași recognize the importance of soft skills for their future professional practice, with ethical and professional values, artistic skills, and empathy being most frequently rated as “very important.” The statistically significant differences identified by gender—in favor of female students for most of the skill categories investigated, including empathy—confirm a consistent pattern that is also reported in the international specialized literature.

Multivariate cluster analysis identified four distinct student typologies—high-overall profile, pragmatic-managerial profile, low-overall profile, and moderate-creative profile, respectively—each exhibiting statistically significant differences in demographic structure, professional motivations, and preferences for future practice.

Overall, the results of this study reaffirm the need to assign soft skills a key role in the training of future dentists, as a complement to specialized clinical training. Contemporary dentistry requires more than technical excellence: it requires the ability to understand the patient, communicate effectively, build relationships based on trust, and act with ethical responsibility. Strengthening these skills as early as the undergraduate training period thus becomes a fundamental component of preparing professionals capable of providing not only high-quality dental care but also truly patient-centered care.

## Figures and Tables

**Table 1:** The qualitative evaluation of soft skills importance scores in the global sample.

The Importance of Soft Skills Main Categories:	Qualitative Evaluation											
	Very Low		Low		Moderate		High		Very High		Total	
	n	%	n	%	n	%	n	%	n	%	n	%
Cognitive Skills	0	0.0	8	3.9	62	30.2	92	44.9	43	21.0	205	100.0
Communication and Interpersonal Skills	0	0.0	1	0.5	27	13.2	131	63.9	46	22.4	205	100.0
Artistic Skills	2	1.0	9	4.4	30	14.6	49	23.9	115	56.1	205	100.0
Ethical and Professional Values	0	0.0	1	0.5	10	4.9	69	33.7	125	61.0	205	100.0
Personal Characteristics	1	0.5	4	2.0	52	25.4	71	34.6	77	37.6	205	100.0
Management Skills	0	0.0	2	1.0	68	33.2	79	38.5	56	27.3	205	100.0
Empathy	7	3.4	7	3.4	32	15.6	38	18.5	121	59.0	205	100.0

**Table 2:** The quantitative evaluation of soft skills importance scores in the global sample.

The Importance of Soft Skills Main Categories:	Quantitative Evaluation			
	m ± SD	Min ÷ Max	Median	IQR
Cognitive Skills	10.57 ± 2.303	4 ÷ 15	11.00	9.00 ÷ 12.00
Communication and Interpersonal Skills	25.60 ± 3.815	14 ÷ 33	26.00	23.00 ÷ 28.00
Artistic Skills	4.30 ± 0.942	1 ÷ 5	5.00	4.00 ÷ 5.00
Ethical and Professional Values	20.90 ± 3.105	10 ÷ 25	21.00	19.00 ÷ 23.00
Personal Characteristics	7.69 ± 1.720	2 ÷ 10	8.00	6.00 ÷ 9.00
Management Skills	7.43 ± 1.512	4 ÷ 10	7.00	6.00 ÷ 9.00
Empathy	4.26 ± 1.061	1 ÷ 5	5.00	4.00 ÷ 5.00

**Table 3:** The quantitative characterization of the identified clusters.

	Initial Cluster Centers				Final Cluster Centers			
	1 (n = 75)	2 (n = 36)	3 (n = 12)	4 (n = 82)	1 (n = 75)	2 (n = 36)	3 (n = 12)	4 (n = 82)
Z-score: Importance of cognitive skills	1.055	−0.682	−1.116	0.186	0.690	−0.043	−0.754	−0.502
Z-score: Importance of communication skills	1.679	0.106	−3.039	−1.991	0.802	0.179	−1.488	−0.594
Z-score: Importance of artistic skills	0.746	−1.378	−2.440	−1.378	0.576	−1.526	−1.201	0.319
Z-score: Importance of ethical-professional values	0.999	−3.188	−3.188	−0.289	0.737	−0.387	−1.899	−0.226
Z-score: Importance of personal features	1.341	−0.984	0.760	−3.309	0.589	−0.370	−1.275	−0.190
Z-score: Importance of management skills	−0.945	1.700	−2.268	−1.607	0.342	0.653	−1.221	−0.421

**Table 4:** The comparative study of the final identified clusters: demographic features.

		Final Identified Clusters								*p*-Value ^†^
		1 (High Global Profile)		2 (Pragmatic-Management Profile)		3 (Low Global Profile)		4 (Moderate-Creative Profile)		
		n	%	n	%	n	%	n	%	
Gender	male	18	24.0%	11	30.6%	9	75.0%	37	45.1%	0.001 **
	female	57	76.0%	25	69.4%	3	25.0%	45	54.9%	
Age	≤22 years	24	32.0%	9	25.0%	3	25.0%	25	30.5%	0.794
	23—24 years	37	49.3%	23	63.9%	8	66.7%	44	53.7%	
	≥25 years	14	18.7%	4	11.1%	1	8.3%	13	15.9%	
Series	Romanian	52	69.3%	21	58.3%	6	50.0%	57	69.5%	0.371
	Abroad	23	30.7%	15	41.7%	6	50.0%	25	30.5%	
Total		75	100.0%	36	100.0%	12	100.0%	82	100.0%	

^†^ Pearson Chi-squared test; ** *p* < 0.01 statistically highly significant.

**Table 5:** The comparative study of the final identified clusters according to the students’ motivations to choose the career of stomatologist, requirements for supplementary knowledges additional to the specialty preparation, and preferences regarding the environment of the future practice.

		Final Identified Clusters								*p*-Value
		1 (High Global Profile)		2 (Pragmatic-Management Profile)		3 (Low Global Profile)		4 (Moderate-Creative Profile)		
		n	%	n	%	n	%	n	%	
motivation	M1	52	69.3%	26	72.2%	6	50.0%	55	67.1%	0.539
	M2	24	32.0%	8	22.2%	1	8.3%	16	19.5%	0.156
	M3	58	77.3%	31	86.1%	11	91.7%	69	84.1%	0.465
	M4	44	58.7%	26	72.2%	9	75.0%	66	80.5%	0.027 *
	M5	48	64.0%	18	50.0%	4	33.3%	49	59.8%	0.161
	M6	26	34.7%	7	19.4%	1	8.3%	23	28.0%	0.152
skills	C1	64	85.3%	30	83.3%	7	58.3%	71	86.6%	0.095
	C2	56	74.7%	28	77.8%	4	33.3%	63	76.8%	0.013 *
	C3	57	76.0%	23	63.9%	4	33.3%	56	68.3%	0.027 *
	C4	57	76.0%	24	66.7%	11	91.7%	64	78.0%	0.317
	C5	59	78.7%	24	66.7%	6	50.0%	58	70.7%	0.167
performances	P1	30	40.0%	29	80.6%	5	41.7%	43	52.4%	<0.001 **
	P2	60	80.0%	19	52.8%	8	66.7%	67	81.7%	0.005 **
	P3	24	32.0%	5	13.9%	2	16.7%	18	22.0%	0.157
	P4	8	10.7%	2	5.6%	2	16.7%	4	4.9%	0.335
Total		75	100.0%	36	100.0%	12	100.0%	82	100.0%	

* *p* < 0.05 statistically significant; ** *p* < 0.01 statistically highly significant.

**Table 6:** The comparative study of the final identified clusters: students’ opinions regarding the importance of empathy in their future practice.

		Final Identified Clusters								*p*-Value
		1 (High Global Profile)		2 (Pragmatic-Management Profile)		3 (Low Global Profile)		4 (Moderate-Creative Profile)		
		n	%	n	%	n	%	n	%	
Empathy importance	very low	3	4.0%	0	0.0%	3	25.0%	1	1.2%	<0.001 **
	low	1	1.3%	2	5.6%	0	0.0%	4	4.9%	
	moderate	3	4.0%	2	5.6%	6	50.0%	21	25.6%	
	high	11	14.7%	6	16.7%	1	8.3%	20	24.4%	
	very high	57	76.0%	26	72.2%	2	16.7%	36	43.9%	
Total		75	100.0%	36	100.0%	12	100.0%	82	100.0%	

** *p* < 0.01 statistically highly significant.

## Data Availability

The data presented in this study are available on request from the 613 corresponding author. The data are not publicly available due to privacy restrictions.

## Appendix A

**Table A1:** The items included in the Motivation, Skills, and Preferences domains of the questionnaire.

Motivation to choose a career in Dentistry	M1: I want to work in a prestigious and respectable profession
	M2: I want to work as part of a team
	M3: I can be a freelancer or independent professional and have flexible working hours
	M4: Dentistry offers financial stability
	M5: Dentistry is a caring and noble profession
	M6: Dentistry offers many social opportunities
Additional skills required in practice	C1: Medical Ethics
	C2: Health Care Law
	C3: Behavioral Sciences
	C4: Health Care Management and Marketing
	C5: Dental Ergonomics
	C6: Others
Preferences for working environment	P1: In a single-dentist practice
	P2: In a large dental clinic
	P3: As a university professor
	P4: As a researcher in the field of Dentistry
	P5: Other

## Appendix B

**Table A2:** The qualitative evaluation of soft skills importance scores—comparative study on genders.

		Gender				*p*-Value ^†^
		Male		Female		
		n	%	n	%	
The Importance of Cognitive Skills	low	3	4.0%	5	3.8%	0.408
	moderate	28	37.3%	34	26.2%	
	high	30	40.0%	62	47.7%	
	very high	14	18.7%	29	22.3%	
The Importance of Communication and Interpersonal Skills	low	1	1.3%	0	0.0%	<0.001 **
	moderate	17	22.7%	10	7.7%	
	high	49	65.3%	82	63.1%	
	very high	8	10.7%	38	29.2%	
The Importance of Artistic Skills	very low	0	0.0%	2	1.5%	0.245
	low	5	6.7%	4	3.1%	
	moderate	8	10.7%	22	16.9%	
	high	22	29.3%	27	20.8%	
	very high	40	53.3%	75	57.7%	
The Importance of Ethical and Professional Values	low	0	0.0%	1	0.8%	<0.001 **
	moderate	7	9.3%	3	2.3%	
	high	35	46.7%	34	26.2%	
	very high	33	44.0%	92	70.8%	
The Importance of Personal Characteristics	very low	1	1.3%	0	0.0%	0.017 *
	low	2	2.7%	2	1.5%	
	moderate	27	36.0%	25	19.2%	
	high	26	34.7%	45	34.6%	
	very high	19	25.3%	58	44.6%	
The Importance of Management Skills	low	1	1.3%	1	0.8%	0.162
	moderate	32	42.7%	36	27.7%	
	high	25	33.3%	54	41.5%	
	very high	17	22.7%	39	30.0%	
The Importance of EMPATHY	very low	7	9.3%	0	0.0%	<0.001 **
	low	2	2.7%	5	3.8%	
	moderate	17	22.7%	15	11.5%	
	high	16	21.3%	22	16.9%	
	very high	33	44.0%	88	67.7%	
Total		75	100.0%	130	100.0%	

^†^ Pearson Chi-squared test; * *p* < 0.05 statistically significant; ** *p* < 0.01 statistically highly significant.

**Table A3:** The qualitative evaluation of soft skills importance scores—comparative study on nationalities.

		Series				*p*-Value ^†^
		Romanian		Abroad		
		n	%	n	%	
The Importance of Cognitive Skills	low	7	5.1%	1	1.4%	0.153
	moderate	39	28.7%	23	33.3%	
	high	66	48.5%	26	37.7%	
	very high	24	17.6%	19	27.5%	
The Importance of Communication and Interpersonal Skills	low	0	0.0%	1	1.4%	0.282
	moderate	20	14.7%	7	10.1%	
	high	83	61.0%	48	69.6%	
	very high	33	24.3%	13	18.8%	
The Importance of Artistic Skills	very low	1	0.7%	1	1.4%	0.159
	low	7	5.1%	2	2.9%	
	moderate	17	12.5%	13	18.8%	
	high	39	28.7%	10	14.5%	
	very high	72	52.9%	43	62.3%	
The Importance of Ethical and Professional Values	low	0	0.0%	1	1.4%	0.009 **
	moderate	4	2.9%	6	8.7%	
	high	39	28.7%	30	43.5%	
	very high	93	68.4%	32	46.4%	
The Importance of Personal Characteristics	very low	0	0.0%	1	1.4%	0.117
	low	3	2.2%	1	1.4%	
	moderate	28	20.6%	24	34.8%	
	high	49	36.0%	22	31.9%	
	very high	56	41.2%	21	30.4%	
The Importance of Management Skills	low	0	0.0%	2	2.9%	<0.001 **
	moderate	31	22.8%	37	53.6%	
	high	67	49.3%	12	17.4%	
	very high	38	27.9%	18	26.1%	
The Importance of EMPATHY	very low	2	1.5%	5	7.2%	0.096
	low	4	2.9%	3	4.3%	
	moderate	18	13.2%	14	20.3%	
	high	25	18.4%	13	18.8%	
	very high	87	64.0%	34	49.3%	
Total		136	100.0%	69	100.0%	

^†^ Pearson Chi-squared test; ** *p* < 0.01 statistically highly significant.

**Table A4:** The qualitative evaluation of soft skills importance scores—comparative study on age groups.

		Age						*p*-Value ^†^
		≤22 Years		23–24 Years		≥25 Years		
		n	%	n	%	n	%	
The Importance of Cognitive Skills	low	0	0.0%	7	6.3%	1	3.1%	0.313
	moderate	23	37.7%	31	27.7%	8	25.0%	
	high	25	41.0%	53	47.3%	14	43.8%	
	very high	13	21.3%	21	18.8%	9	28.1%	
The Importance of Communication and Interpersonal Skills	low	0	0.0%	1	0.9%	0	0.0%	0.470
	moderate	5	8.2%	18	16.1%	4	12.5%	
	high	40	65.6%	73	65.2%	18	56.3%	
	very high	16	26.2%	20	17.9%	10	31.3%	
The Importance of Artistic Skills	very low	1	1.6%	0	0.0%	1	3.1%	0.282
	low	1	1.6%	8	7.1%	0	0.0%	
	moderate	9	14.8%	18	16.1%	3	9.4%	
	high	15	24.6%	28	25.0%	6	18.8%	
	very high	35	57.4%	58	51.8%	22	68.8%	
The Importance of Ethical and Professional Values	low	1	1.6%	0	0.0%	0	0.0%	0.249
	moderate	1	1.6%	6	5.4%	3	9.4%	
	high	26	42.6%	34	30.4%	9	28.1%	
	very high	33	54.1%	72	64.3%	20	62.5%	
The Importance of Personal Characteristics	very low	0	0.0%	1	0.9%	0	0.0%	0.359
	low	3	4.9%	1	0.9%	0	0.0%	
	moderate	12	19.7%	33	29.5%	7	21.9%	
	high	21	34.4%	35	31.3%	15	46.9%	
	very high	25	41.0%	42	37.5%	10	31.3%	
The Importance of Management Skills	low	1	1.6%	1	0.9%	0	0.0%	0.494
	moderate	15	24.6%	40	35.7%	13	40.6%	
	high	23	37.7%	45	40.2%	11	34.4%	
	very high	22	36.1%	26	23.2%	8	25.0%	
The Importance of EMPATHY	very low	1	1.6%	5	4.5%	1	3.1%	0.785
	low	2	3.3%	5	4.5%	0	0.0%	
	moderate	9	14.8%	16	14.3%	7	21.9%	
	high	14	23.0%	20	17.9%	4	12.5%	
	very high	35	57.4%	66	58.9%	20	62.5%	
Total		61	100.0%	112	100.0%	32	100.0%	

^†^ Pearson Chi-squared test.

**Table A5:** The qualitative evaluation of soft skills importance scores—comparative study by the students’ motivations to choose the career of stomatologist.

		M1: No/Yes n(%)	M2: No/Yes n(%)	M3: No/Yes n(%)	M4: No/Yes n(%)	M5: No/Yes n(%)	M6: No/Yes n(%)
The Importance of Cognitive Skills	low	3 (4.5%)/ 5 (3.6%)	7 (4.5%)/ 1 (2.0%)	1 (2.8%)/ 7 (4.1%)	2 (3.3%)/ 6 (4.1%)	3 (3.5%)/ 5 (4.2%)	7 (4.7%)/ 1 (1.8%)
	moderate	22 (33.3%)/40 (28.8%)	50 (32.1%)/12 (24.5%)	9 (25.0%)/ 53 (31.4%)	11 (18.3%)/51 (35.2%)	30 (34.9%)/32 (26.9%)	43 (29.1%)/19 (33.3%)
	high	31 (47.0%)/61 (43.9%)	71 (45.5%)/21 (42.9%)	17 (47.2%)/75 (44.4%)	27 (45.0%)/65 (44.8%)	37 (43.0%)/55 (46.2%)	64 (43.2%)/28 (49.1%)
	very high	10 (15.2%)/33 (23.7%)	28 (17.9%)/15 (30.6%)	9 (25.0%)/ 34 (20.1%)	20 (33.3%)/23 (15.9%)	16 (18.6%)/27 (22.7%)	34 (23.0%)/ 9 (15.8%)
*p*-value		0.556	0.242	0.818	0.017 *	0.653	0.473
The Importance of Communication and Interpersonal Skills	low	-/ 1 (0.7%)	1 (0.6%)/ -	1 (2.8%)/ -	1 (1.7%)/ -	-/ 1 (0.8%)	1 (0.7%)/ -
	moderate	12 (18.2%)/15 (10.8%)	21 (13.5%)/ 6 (12.2%)	5 (13.9%)/ 22 (13.0%)	6 (10.0%)/ 21 (14.5%)	11 (12.8%)/16 (13.4%)	24 (16.2%)/ 3 (5.3%)
	high	36 (54.5%)/95 (68.3%)	100 (64.1%)/31 (63.3%)	22 (61.1%)/109 (64.5%)	40 (66.7%)/91 (62.8%)	55 (64.0%)/76 (63.9%)	95 (64.2%)/36 (63.2%)
	very high	18 (27.3%)/28 (20.1%)	34 (21.8%)/12 (24.5%)	8 (22.2%)/ 38 (22.5%)	13 (21.7%)/33 (22.8%)	20 (23.3%)/26 (21.8%)	28 (18.9%)/18 (31.6%)
*p*-value		0.197	0.923	0.190	0.364	0.854	0.069
The Importance of Artistic Skills	very low	1 (1.5%)/ 1 (0.7%)	2 (1.3%)/ -	-/ 2 (1.2%)	-/ 2 (1.4%)	1 (1.2%)/ 1 (0.8%)	2 (1.4%)/ -
	low	4 (6.1%)/ 5 (3.6%)	7 (4.5%)/ 2 (4.1%)	1 (2.8%)/ 8 (4.7%)	5 (8.3%)/ 4 (2.8%)	4 (4.7%)/ 5 (4.2%)	7 (4.7%)/ 2 (3.5%)
	moderate	8 (12.1%)/ 22 (15.8%)	22 (14.1%)/ 8 (16.3%)	4 (11.1%)/ 26 (15.4%)	8 (13.3%)/22 (15.2%)	16 (18.6%)/14 (11.8%)	27 (18.2%)/ 3 (5.3%)
	high	16 (24.2%)/33 (23.7%)	37 (23.7%)/12 (24.5%)	11 (30.6%)/38 (22.5%)	9 (15.0%)/40 (27.6%)	20 (23.3%)/29 (24.4%)	35 (23.6%)/ 14 (24.6%)
	very high	37 (56.1%)/78 (56.1%)	88 (56.4%)/27 (55.1%)	20 (55.6%)/95 (56.2%)	38 (63.3%)/77 (53.1%)	45 (52.3%)/70 (58.8%)	77 (52.0%)/ 38 (66.7%)
*p*-value		0.856	0.940	0.760	0.110	0.722	0.124
The Importance of Ethical and Professional Values	low	-/ 1 (0.7%)	1 (0.6%)/ -	-/ 1 (0.6%)	-/ 1 (0.7%)	1 (1.2%)/ -	1 (0.7%)/ -
	moderate	4 (6.1%)/ 6 (4.3%)	8 (5.1%)/ 2 (4.1%)	1 (2.8%)/ 9 (5.3%)	3 (5.0%)/ 7 (4.8%)	5 (5.8%)/ 5 (4.2%)	9 (6.1%)/ 1 (1.8%)
	high	23 (34.8%)/46 (33.1%)	55 (35.3%)/14 (28.6%)	9 (25.0%)/ 60 (35.5%)	14 (23.3%)/55 (37.9%)	31 (36.0%)/ 38 (31.9%)	52 (35.1%)/ 17 (29.8%)
	very high	39 (59.1%)/86 (61.9%)	92 (59.0%)/33(67.3%)	26 (72.2%)/99 (58.6%)	43 (71.7%)/82 (56.6%)	49 (57.0%)/76 (63.9%)	86 (58.1%)/39 (68.4%)
*p*-value		0.837	0.724	0.478	0.196	0.515	0.387
The Importance of Personal Characteristics	very low	1 (1.5%)/ -	1 (0.6%)/ -	-/ 1 (0.6%)	-/1 (0.7%)	1 (1.2%)/ -	1 (0.7%)/ -
	low	2 (3.0%)/ 2 (1.4%)	3 (1.9%)/ 1 (2.0%)	1 (2.8%)/ 3 (1.8%)	-/4 (2.8%)	3 (3.5%)/ 1 (0.8%)	4 (2.7%)/ -
	moderate	18 (27.3%)/34 (24.5%)	42 (26.9%)/10 (20.4%)	6 (16.7%)/ 46 (27.2%)	13 (21.7%)/39 (26.9%)	29 (33.7%)/23 (19.3%)	37 (25.0%)/15 (26.3%)
	high	18 (27.3%)/53 (38.1%)	56 (35.9%)/15 (30.6%)	12 (33.3%)/59 (34.9%)	19 (31.7%)/52 (35.9%)	26 (30.2%)/45 (37.8%)	53 (35.8%)/18 (31.6%)
	very high	27 (40.9%)/50 (36.0%)	54 (34.6%)/23 (46.9%)	17 (47.2%)/60 (35.5%)	28 (46.7%)/49 (33.8%)	27 (31.4%)/50 (42.0%)	53 (35.8%)/24 (42.1%)
*p*-value		0.326	0.601	0.596	0.329	0.048 *	0.627
The Importance of Management Skills	low	-/ 2 (1.4%)	2 (1.3%)/ -	1 (2.8%)/ 1 (0.6%)	2 (3.3%)/ -	-/2 (1.7%)	2 (1.4%)/ -
	moderate	24 (36.4%)/ 44 (31.7%)	51 (32.7%)/ 17 (34.7%)	8 (22.2%)/ 60 (35.5%)	14 (23.3%)/54 (37.2%)	24 (27.9%)/44 (37.0%)	49 (33.1%)/19 (33.3%)
	high	24 (36.4%)/55 (39.6%)	62 (39.7%)/ 17 (34.7%)	16 (44.4%)/63 (37.3%)	23 (38.3%)/56 (38.6%)	41 (47.7%)/38 (31.9%)	56 (37.8%)/23 (40.4%)
	very high	18 (27.3%)/38 (27.3%)	41 (26.3%)/ 15 (30.6%)	11 (30.6%)/45 (26.6%)	21 (35.0%)/35 (24.1%)	21 (24.4%)/35 (29.4%)	41 (27.7%)/15 (26.3%)
*p*-value		0.713	0.759	0.309	0.027 *	0.096	0.833
The Importance of EMPATHY	very low	3 (4.5%)/ 4 (2.9%)	4 (2.6%)/ 3 (6.1%)	2 (5.6%)/ 5 (3.0%)	4 (6.7%)/ 3 (2.1%)	4 (4.7%)/ 3 (2.5%)	6 (4.1%)/ 1 (1.8%)
	low	3 (4.5%)/ 4 (2.9%)	4 (2.6%)/ 3 (6.1%)	2 (5.6%)/ 5 (3.0%)	3 (5.0%)/ 4 (2.8%)	2 (2.3%)/ 5 (4.2%)	6 (4.1%)/ 1 (1.8%)
	moderate	10 (15.2%)/22 (15.8%)	26 (16.7%)/ 6 (12.2%)	4 (11.1%)/ 28 (16.6%)	4 (6.7%)/ 28 (19.3%)	9 (10.5%)/ 23 (19.3%)	23 (15.5%)/ 9 (15.8%)
	high	12 (18.2%)/26 (18.7%)	31 (19.9%)/ 7 (14.3%)	6 (16.7%)/ 32 (18.9%)	14 (23.3%)/24 (16.6%)	19 (22.1%)/19 (16.0%)	28 (18.9%)/10 (17.5%)
	very high	38 (57.6%)/83 (59.7%)	91 (58.3%)/ 30 (61.2%)	22 (61.1%)/99 (58.6%)	35 (58.3%)/86 (59.3%)	52 (60.5%)/69 (58.0%)	85 (57.4%)/36 (63.2%)
*p*-value		0.941	0.418	0.763	0.070	0.314	0.818
Total		66 (100.0%)/139 (100.0%)	156 (100.0%)/49 (100.0%)	36 (100.0%)/169 (100.0%)	60 (100.0%)/145 (100.0%)	86 (100.0%)/119 (100.0%)	148 (100.0%)/57 (100.0%)

* *p* < 0.05 statistically significant.

**Table A6:** The qualitative evaluation of soft skills importance scores—comparative study by the students’ requirements for supplementary knowledges additional to the specialty preparation.

		C1: No/Yes n(%)	C2: No/Yes n(%)	C3: No/Yes n(%)	C4: No/Yes n(%)	C5: No/Yes n(%)
The Importance of Cognitive Skills	low	2 (6.1%)/ 6 (3.5%)	1 (1.9%)/ 7 (4.6%)	2 (3.1%)/ 6 (4.3%)	1 (2.0%)/ 7 (4.5%)	3 (5.2%)/ 5 (3.4%)
	moderate	13 (39.4%)/ 49 (28.5%)	17 (31.5%)/ 45 (29.8%)	20 (30.8%)/ 42 (30.0%)	13 (26.5%)/ 49 (31.4%)	19 (32.8%)/ 43 (29.3%)
	high	12 (36.4%) 80 (46.5%)	21 (38.9%)/ 71 (47.0%)	27 (41.5%)/ 65 (46.4%)	23 (46.9%)/ 69 (44.2%)	23 (39.7%)/ 69 (46.9%)
	very high	6 (18.2%)/ 37 (21.5%)	15 (27.8%)/ 28 (18.5%)	16 (24.6%)/ 27 (19.3%)	12 (24.5%)/ 31 (19.9%)	13 (22.4%)/ 30 (20.4%)
*p*-value		0.505	0.387	0.798	0.728	0.783
The Importance of Communication and Interpersonal Skills	low	-/ 1 (0.6%)	1 (1.9%)/ -	1 (1.5%)/ -	1 (2.0%)/ -	1 (1.7%)/ -
	moderate	5 (15.2%)/ 22 (12.8%)	8 (14.8%)/ 19 (12.6%)	9 (13.8%)/ 18 (12.9%)	5 (10.2%)/ 22 (14.1%)	11 (19.0%)/ 16 (10.9%)
	high	18 (54.5%)/ 113 (65.7%)	35 (64.8%)/ 96 (63.6%)	46 (70.8%)/ 85 (60.7%)	31 (63.3%)/ 100 (64.1%)	33 (56.9%)/ 98 (66.7%)
	very high	10 (30.3%)/ 36 (20.9%)	10 (18.5%)/ 36 (23.8%)	9 (13.8%)/ 37 (26.4%)	12 (24.5%)/ 34 (21.8%)	13 (22.4%)/ 33 (22.4%)
*p*-value		0.587	0.326	0.111	0.291	0.157
The Importance of Artistic Skills	very low	1 (3.0%)/ 1 (0.6%)	-/ 2 (1.3%)	-/ 2 (1.4%)	1 (2.0%)/ 1 (0.6%)	-/ 2 (1.4%)
	low	3 (9.1%)/ 6 (3.5%)	5 (9.3%)/ 4 (2.6%)	3 (4.6%)/ 6 (4.3%)	4 (8.2%)/ 5 (3.2%)	6 (10.3%)/ 3 (2.0%)
	moderate	3 (9.1%)/ 27 (15.7%)	8 (14.8%)/ 22 (14.6%)	12 (18.5%)/ 18 (12.9%)	6 (12.2%)/ 24 (15.4%)	9 (15.5%)/ 21 (14.3%)
	high	9 (27.3%)/ 40 (23.3%)	14 (25.9%)/ 35 (23.2%)	17 (26.2%)/ 32 (22.9%)	9 (18.4%)/ 40 (25.6%)	14 (24.1%)/ 35 (23.8%)
	very high	17 (51.5%)/ 98 (57.0%)	27 (50.0%)/ 88 (58.3%)	33 (50.8%)/ 82 (58.6%)	29 (59.2%)/ 86 (55.1%)	29 (50.0%)/ 86 (58.5%)
*p*-value		0.304	0.259	0.631	0.403	0.095
The Importance of Ethical and Professional Values	low	-/ 1 (0.6%)	1 (1.9%)/ -	1 (1.5%)/ -	-/ 1 (0.6%)	-/ 1 (0.7%)
	moderate	4 (12.1%)/ 6 (3.5%)	4 (7.4%)/ 6 (4.0%)	6 (9.2%)/ 4 (2.9%)	2 (4.1%)/ 8 (5.1%)	4 (6.9%)/ 6 (4.1%)
	high	11 (33.3%)/ 58 (33.7%)	21 (38.9%)/ 48 (31.8%)	26 (40.0%)/ 43 (30.7%)	19 (38.8%)/ 50 (32.1%)	23 (39.7%)/ 46 (31.3%)
	very high	18 (54.5%)/ 107 (62.2%)	28 (51.9%)/ 97 (64.2%)	32 (49.2%)/ 93 (66.4%)	28 (57.1%)/ 97 (62.2%)	31 (53.4%)/ 94 (63.9%)
*p*-value		0.196	0.148	0.027 *	0.788	0.443
The Importance of Personal Characteristics	very low	-/ 1 (0.6%)	1 (1.9%)/ -	-/ 1 (0.7%)	-/ 1 (0.6%)	1 (1.7%)/ -
	low	1 (3.0%)/ 3 (1.7%)	2 (3.7%)/ 2 (1.3%)	3 (4.6%)/ 1 (0.7%)	-/ 4 (2.6%)	3 (5.2%)/ 1 (0.7%)
	moderate	13 (39.4%)/ 39 (22.7%)	16 (29.6%)/ 36 (23.8%)	23 (35.4%)/ 29 (20.7%)	12 (24.5%)/ 40 (25.6%)	18 (31.0%)/ 34 (23.1%)
	high	11 (33.3%)/ 60 (34.9%)	18 (33.3%)/ 53 (35.1%)	22 (33.8%)/ 49 (35.0%)	19 (38.8%)/ 52 (33.3%)	21 (36.2%)/ 50 (34.0%)
	very high	8 (24.2%)/ 69 (40.1%)	17 (31.5%)/ 60 (39.7%)	17 (26.2%)/ 60 (42.9%)	18 (36.7%)/ 59 (37.8%)	15 (25.9%)/ 62 (42.2%)
*p*-value		0.253	0.264	0.026 *	0.750	0.028 *
The Importance of Management Skills	low	-/ 2 (1.2%)	2 (3.7%)/ -	1 (1.5%)/ 1 (0.7%)	1 (2.0%)/ 1 (0.6%)	2 (3.4%)/ -
	moderate	13 (39.4%)/ 55 (32.0%)	16 (29.6%)/ 52 (34.4%)	19 (29.2%)/ 49 (35.0%)	17 (34.7%)/ 51 (32.7%)	12 (20.7%)/ 56 (38.1%)
	high	11 (33.3%)/ 68 (39.5%)	22 (40.7%)/ 57 (37.7%)	24 (36.9%)/ 55 (39.3%)	19 (38.8%)/ 60 (38.5%)	26 (44.8%)/ 53 (36.1%)
	very high	9 (27.3%)/ 47 (27.3%)	14 (25.9%)/ 42 (27.8%)	21 (32.3%)/ 35 (25.0%)	12 (24.5%)/ 44 (28.2%)	18 (31.0%)/ 38 (25.9%)
*p*-value		0.772	0.111	0.640	0.805	0.018 *
The Importance of EMPATHY	very low	2 (6.1%)/ 5 (2.9%)	4 (7.4%)/ 3 (2.0%)	4 (6.2%)/ 3 (2.1%)	1 (2.0%)/ 6 (3.8%)	3 (5.2%)/ 4 (2.7%)
	low	-/ 7 (4.1%)	2 (3.7%)/ 5 (3.3%)	2 (3.1%)/ 5 (3.6%)	1 (2.0%)/ 6 (3.8%)	3 (5.2%)/ 4 (2.7%)
	moderate	6 (18.2%)/ 26 (15.1%)	8 (14.8%)/ 24 (15.9%)	12 (18.5%)/ 20 (14.3%)	10 (20.4%)/ 22 (14.1%)	5 (8.6%)/ 27 (18.4%)
	high	6 (18.2%)/ 32 (18.6%)	12 (22.2%)/ 26 (17.2%)	14 (21.5%)/ 24 (17.1%)	7 (14.3%)/ 31 (19.9%)	12 (20.7%)/ 26 (17.7%)
	very high	19 (57.6%)/ 102 (59.3%)	28 (51.9%)/ 93 (61.6%)	33 (50.8%)/ 88 (62.9%)	30 (61.2%)/ 91 (58.3%)	35 (60.3%)/ 86 (58.5%)
*p*-value		0.675	0.326	0.382	0.673	0.377
Total		33 (100.0%)/172 (100.0%)	54 (100.0%)/ 151 (100.0%)	65 (100.0%)/140 (100.0%)	49 (100.0%)/156 (100.0%)	58 (100.0%)/147 (100.0%)

* *p* < 0.05 statistically significant.

**Table A7:** The qualitative evaluation of soft skills importance scores—comparative study by the students’ preferences regarding the environment of the future practice.

		P1: No/Yes n(%)	P2: No/Yes n(%)	P3: No/Yes n(%)	P4: No/Yes n(%)
The Importance of Cognitive Skills	low	2 (2.0%)/ 6 (5.6%)	1 (2.0%)/ 7 (4.5%)	6 (3.8%)/ 2 (4.1%)	7 (3.7)/ 1 (6.3%)
	moderate	27 (27.6%)/ 35 (32.7%)	15 (29.4%)/ 47 (30.5%)	50 (32.1%)/ 12 (24.5%)	57 (30.2%)/ 5 (31.3%)
	high	40 (40.8%)/ 52 (48.6%)	24 (47.1%)/ 68 (44.2%)	75 (48.1%)/ 17 (34.7%)	88 (46.6%)/ 4 (25.0%)
	very high	29 (29.6%)/ 14 (13.1%)	11 (21.6%)/ 32 (20.8%)	25 (16.0%)/ 18 (36.7%)	37 (19.6%)/ 6 (37.5%)
*p*-value		0.024 *	0.860	0.020 *	0.257
The Importance of Communication and Interpersonal Skills	low	1 (1.0%)/ -	-/ 1 (0.6%)	1 (0.6%)/ -	1 (0.5%)/ -
	moderate	9 (9.2%)/ 18 (16.8%)	7 (13.7%)/ 20 (13.0%)	21 (13.5%)/ 6 (12.2%)	26 (13.8%)/ 1 (6.3%)
	high	63 (64.3%)/ 68 (63.6%)	33 (64.7%)/ 98 (63.6%)	99 (63.5%)/ 32 (65.3%)	119 (63.0%)/ 12 (75.0%)
	very high	25 (25.5%)/ 21 (19.6%)	11 (21.6%)/ 35 (22.7%)	35 (22.4%)/ 11 (22.4%)	43 (22.8%)/ 3 (18.8%)
*p*-value		0.246	0.945	0.945	0.764
The Importance of Artistic Skills	very low	1 (1.0%)/ 1 (0.9%)	-/ 2 (1.3%)	2 (1.3%)/ -	2 (1.1%)/ -
	low	2 (2.0%)/ 7 (6.5%)	4 (7.8%)/ 5 (3.2%)	8 (5.1%)/ 1 (2.0%)	8 (4.2%)/ 1 (6.3%)
	moderate	9 (9.2%)/ 21 (19.6%)	14 (27.5%)/ 16 (10.4%)	23 (14.7%)/ 7 (14.3%)	28 (14.8%)/ 2 (12.5%)
	high	22 (22.4%)/ 27 (25.2%)	12 (23.5%)/ 37 (24.0%)	42 (26.9%)/ 7 (14.3%)	45 (23.8%)/ 4 (25.0%)
	very high	64 (65.3%)/ 51 (47.7%)	21 (41.2%)/ 94 (61.0%)	81 (51.9%)/ 34 (69.4%)	106 (56.1%)/ 9 (56.3%)
*p*-value		0.057	0.012 *	0.202	0.985
The Importance of Ethical and Professional Values	low	-/ 1 (0.9%)	-/ 1 (0.6%)	1 (0.6%)/ -	1 (0.5%)/ -
	moderate	7 (7.1%)/ 3 (2.8%)	2 (3.9%)/ 8 (5.2%)	8 (5.1%)/ 2 (4.1%)	10 (5.3%)/ -
	high	24 (24.5%)/ 45 (42.1%)	23 (45.1%)/ 46 (29.9%)	57 (36.5%)/ 12 (24.5%)	64 (33.9%)/ 5 (31.3%)
	very high	67 (68.4%)/ 58 (54.2%)	26 (51.0%)/ 99 (64.3%)	90 (57.7%)/ 35 (71.4%)	114 (60.3%)/ 11 (68.8%)
*p*-value		0.026 *	0.240	0.368	0.769
The Importance of Personal Characteristics	very low	-/ 1 (0.9%)	1 (2.0%)/ -	1 (0.6%)/ -	1 (0.5%)/ -
	low	1 (1.0%)/ 3 (2.8%)	2 (3.9%)/ 2 (1.3%)	4 (2.6%)/ -	4 (2.1%)/ -
	moderate	21 (21.4%)/ 31 (29.0%)	14 (27.5%)/ 38 (24.7%)	41 (26.3%)/ 11 (22.4%)	48 (25.4%)/ 4 (25.0%)
	high	32 (32.7%)/ 39 (36.4%)	19 (37.3%)/ 52 (33.8%)	52 (33.3%)/ 19 (38.8%)	66 (34.9%)/ 5 (31.3%)
	very high	44 (44.9%)/ 33 (30.8)	15 (29.4%)/ 62 (40.3%)	58 (37.2%)/ 19 (38.8%)	70 (37.0%)/ 7 (43.8%)
*p*-value		0.215	0.213	0.712	0.956
The Importance of Management Skills	low	1 (1.0%)/ 1 (0.9%)	-/ 2 (1.3%)	2 (1.3%)/ -	2 (1.1%)/ -
	moderate	31 (31.6%)/ 37 (34.6%)	14 (27.5%)/ 54 (35.1%)	53 (34.0%)/ 15 (30.6%)	63 (33.3%)/ 5 (31.3%)
	high	40 (40.8%)/ 39 (36.4%)	19 (37.3%)/ 60 (39.0%)	58 (37.2%)/ 21 (42.9%)	72 (38.1%)/ 7 (43.8%)
	very high	26 (26.5%)/ 30 (28.0%)	18 (35.3%)/ 38 (24.7%)	43 (27.6%)/ 13 (26.5%)	52 (27.5%)/ 4 (25.0%)
*p*-value		0.933	0.401	0.781	0.951
The Importance of EMPATHY	very low	6 (6.1%)/ 1 (0.9%)	1 (2.0%)/ 6 (3.9%)	4 (2.6%)/ 3 (6.1%)	6 (3.2%)/ 1 (6.3%)
	low	3 (3.1%)/ 4 (3.7%)	1 (2.0%)/ 6 (3.9%)	4 (2.6%)/ 3 (6.1%)	7 (3.7%)/ -
	moderate	13 (13.3%)/ 19 (17.8%)	5 (9.8%)/ 27 (17.5%)	27 (17.3%)/ 5 (10.2%)	29 (15.3%)/ 3 (18.8%)
	high	14 (14.3%)/ 24 (22.4%)	12 (23.5%)/ 26 (16.9%)	32 (20.5%)/ 6 (12.2%)	35 (18.5%)/ 3 (18.8%)
	very high	62 (63.3%)/ 59 (55.1%)	32 (62.7%)/ 89 (57.8%)	89 (57.1%)/ 32 (65.3%)	112 (59.3%)/ 9 (56.3%)
*p*-value		0.127	0.497	0.216	0.889
Total		98 (100.0%)/107 (100.0%)	51 (100.0%)/ 154 (100.0%)	156 (100.0%)/49 (100.0%)	189 (100.0%)/16 (100.0%)

* *p* < 0.05 statistically significant.
